# Effect of unloading brace treatment on pain and function in patients with symptomatic knee osteoarthritis: the ROTOR randomized clinical trial

**DOI:** 10.1038/s41598-018-28782-3

**Published:** 2018-07-12

**Authors:** Philippe Thoumie, Marc Marty, Bernard Avouac, Adeline Pallez, Arnaud Vaumousse, Linh Pham Thi Pipet, André Monroche, Nicolas Graveleau, Armand Bonnin, Cyrine Ben Amor, Emmanuel Coudeyre

**Affiliations:** 1 APHP Hôpital Rothschild and Sorbonne University Agathe INSERM U-1150, Service de Médecine Physique et Réadaptation, Paris, 75012 France; 2 Nukléus, Paris, 75013 France; 3 Hôpital Henri Mondor, Service de Rhumatologie, Créteil, 94000 France; 4 Thuasne, Levallois-Perret, 92300 France; 5 Euraxi Pharma, Joué-les-Tours, 37303 France; 6 Cabinet Médical, Bourges, 18000 France; 7 Cabinet Médical, Angers, 49100 France; 8 Clinique du Sport, Bordeaux, 33700 France; 9 CHU Clermont-Ferrand, Service de Médecine Physique et de Réadaptation, Université Clermont Auvergne, INRA UNH UMR 1019, Clermont-Ferrand, 63003 France

## Abstract

Evidence is still inconclusive for the benefits of bracing in patients with knee osteoarthritis. To assess the effect of REBEL RELIEVER unloading knee brace in conservative treatment of knee osteoarthritis, a randomized controlled trial was conducted in 67 patients with symptomatic medial knee osteoarthritis, who randomly received 6-week treatment with either REBEL RELIVER unloading knee brace + usual care (Brace group, N = 32) or usual care alone (Control group, N = 35). Primary outcome was the global last 24h-pain relief (100-mm visual analogic scale [VAS]) at 6 weeks. Secondary endpoints included pain on motion (100-mm VAS), function (Lequesne index), safety and observance. At 6 weeks, mean [SD] last 24h-pain decreased significantly more in Brace group versus Control group (−41.35 [3.37] vs −15.37 [3.23], difference −25.98, 95% CI −41.64 to −10.33, P < 0.0001). Higher mean [SD] pain on motion decrease (−51.91 [3.49] vs −19.91 [3.34], difference −32.01, 95% CI −48.21 to −15.80, P < 0.0001) and better improvement of Lequesne index score (−5.8 [0.5] vs −2.3 [0.5], difference −3.5, 95% CI −5.0 to −2.0, P < 0.0001) were observed in Brace group. Safety and observance to the brace were excellent. The additive clinical benefit of wearing REBEL RELIEVER unloading knee brace was demonstrated in knee osteoarthritis patients.

## Introduction

Knee osteoarthritis (OA) is a common medical condition that causes considerable pain and immobility, leading to functional disability and impaired quality of life. The prevalence of OA has been reported to increase as the population ages^1,2^. In France, knee OA prevalence was estimated at 4.7% for men and 6.6% for women and ranged from 2.1% to 10.1% for men and 1.6% to 14.9% for women according to age class^3^. Knee OA can affect one (unilateral) or both (bilateral) knees and may involve all three joint compartments. In patients with single-compartment disease, the inner (medial) compartment is the most commonly affected^4^.

According to Osteoarthritis Research Society International (OARSI) guidelines, initial management of knee OA is conservative and consists of a combination of both non-pharmacological and pharmacological treatments, including exercise programs, self-management and education (adaptation of activities and/or weight reduction), biomechanical interventions (knee braces, foot orthoses), and pain killers (analgesics such as acetaminophen, non-steroidal anti-inflammatory drugs [NSAIDs], and intra-articular corticosteroids)^5^.

**Figure 1:**
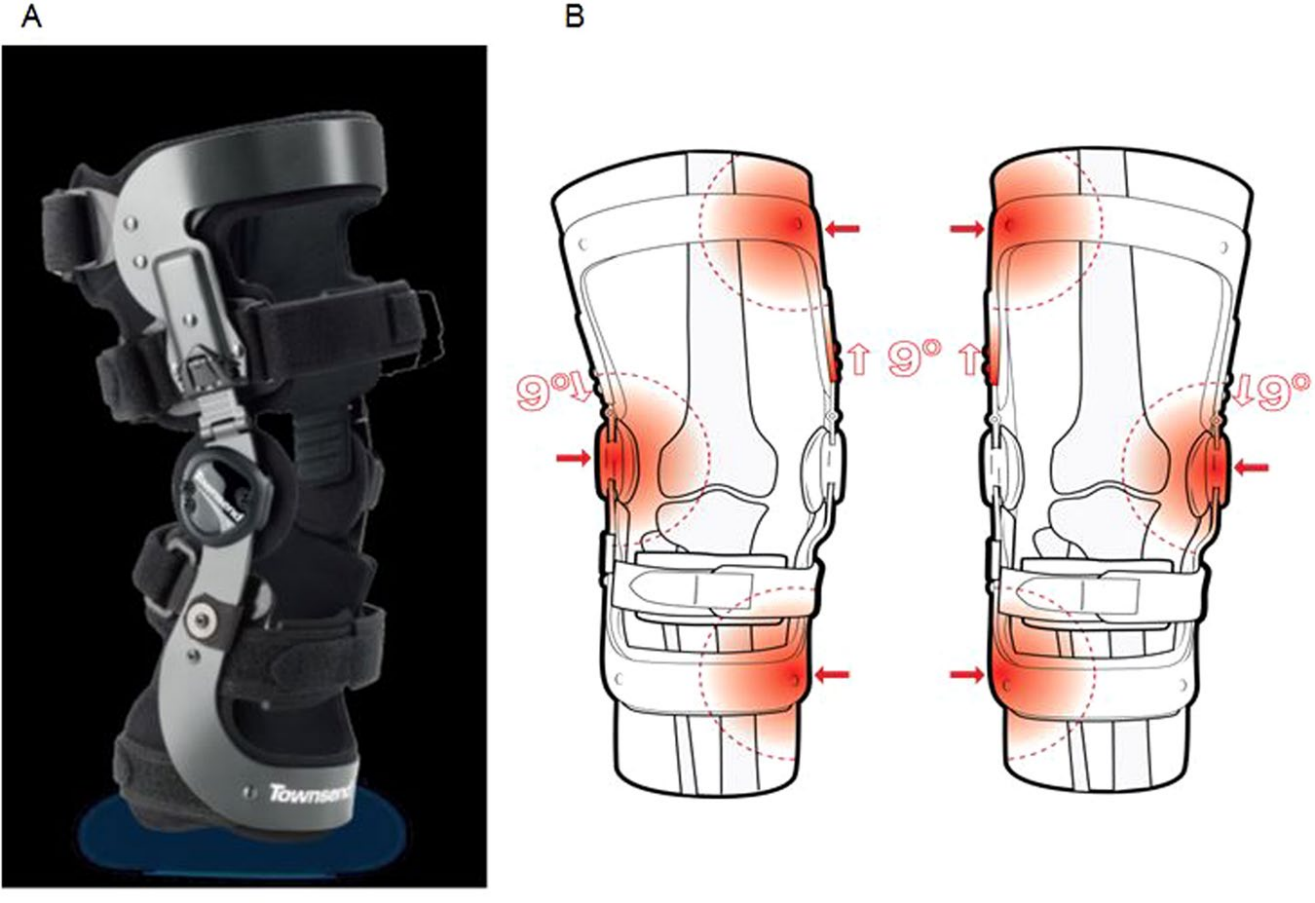
REBEL RELIEVER unloading knee brace picture (**A**) and mechanism of action (**B**). The REBEL RELIEVER unloading knee brace is universally adaptable for medial osteoarthritis (OA), lateral OA, and ligament instability. The brace is designed to stabilize the knee by gently applying a three-point corrective force to the leg. Townsend’s patented LoadShifter technology provides the necessary mechanical leverage to shift compressive forces away from the damaged side of the knee. The rigid superstructure of the brace effectively limits varus or valgus misalignment, and redistributes the patient’s weight across a broader aspect of the knee joint resulting in less pain.

The general purpose of unloading knee braces is to apply corrective forces by means of a three-point pressure system, which distribute load away from the damaged compartment. The applied forces should decrease pain and improve function. Although several studies have investigated the effectiveness of unloading brace treatment for knee OA^6–12^, the recently published Cochrane review that included 13 studies (4 investigating unloading knee braces) concluded that there is still very limited evidence for the benefits of bracing, mainly because of bias questioning the results from most studies^13^. In addition, the reported beneficial effect of unloading knee braces varies depending on whether they are compared to controls without brace, with neutral knee braces, with knee sleeves or with insoles^14^. Therefore, the aim of ROTOR study was to assess, in a randomized controlled trial, the additive effect of the REBEL RELIEVER unloading knee brace, which contains a patented Loadshifter Relief Mechanism for adjusting corrective forces (Fig. 1), in conservative treatment of medial OA. The ROTOR cohort consisted of patients with symptomatic medial knee OA who required management of persistent disabling pain. The REBEL RELIEVER unloading knee brace is designed to accomplish two primary goals. First, the rigid superstructure of the brace will help maintain the leg in a normal (also called neutral) alignment. Second, the brace applies corrective forces by means of a three-point pressure system. These corrective forces help distribute load away from the damaged compartment. The amount of corrective force can be increased as needed by making adjustments to the angle of the thigh shell.

## Results

### Patients

**Figure 2:**
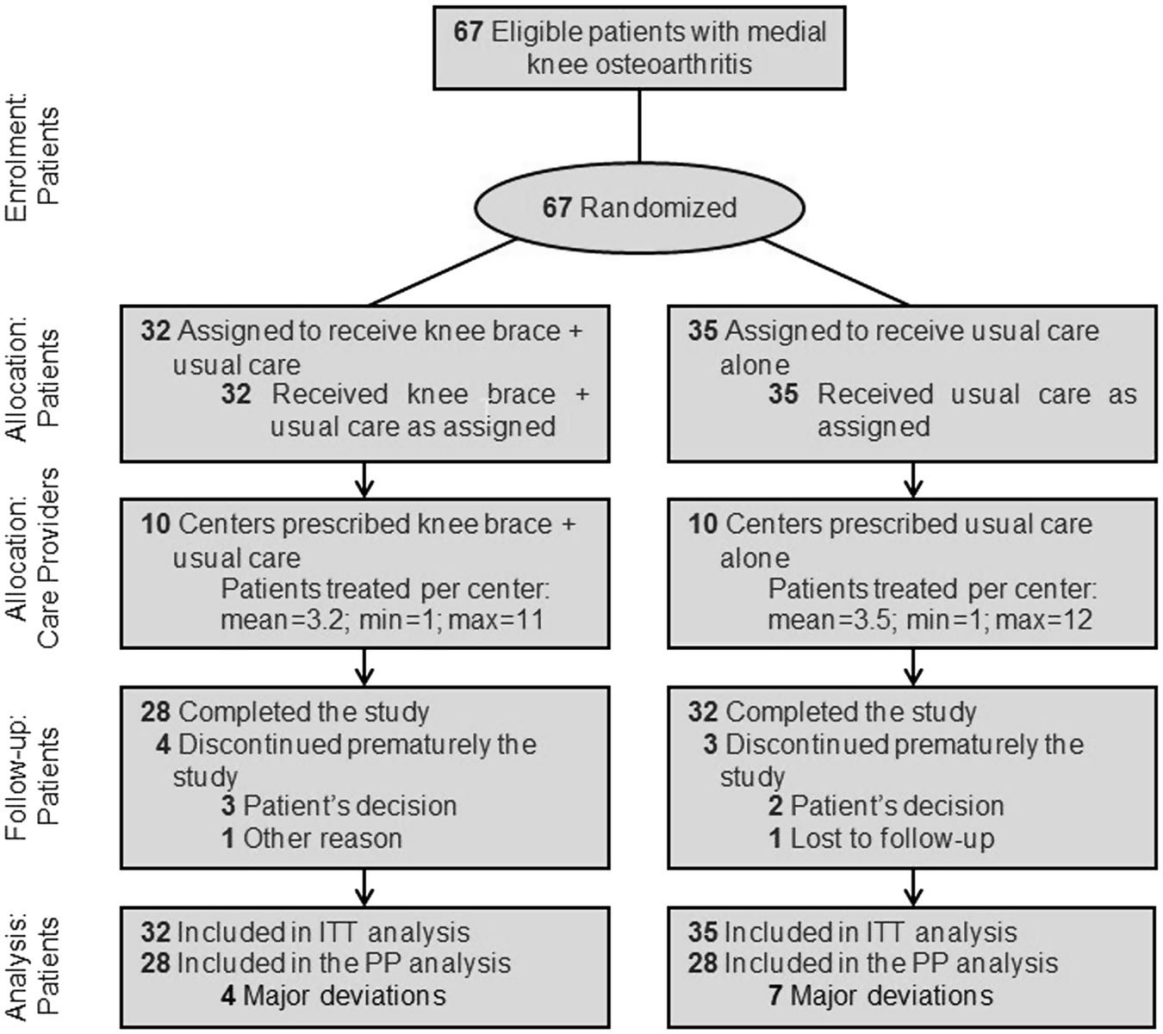
Flowchart of patients in the ROTOR study. ITT: intent-to-treat; Max: maximum; min: minimum; PP: per protocol.

A total of 67 patients were screened for eligibility and randomly assigned to receive either a REBEL RELIEVER unloading knee brace + usual care (n = 32) or usual care alone (n = 35) (Fig. 2). Seven patients withdrew from the study (4 [12.5%] in the Brace group and 3 [8.6%] in the Control group) and 60 patients (89.6%) completed the trial. Reasons for study interruption included patient’s consent withdrawal (5 patients [71.4%]), lost to follow-up (1 patient [14.3%]) and other reason (1 patient [14.3%]). The intent-to-treat (ITT) and safety population sets comprised 67 patients. Seven patients presented major protocol deviations (did not fulfil inclusion/exclusion criteria; duration of treatment <30 days; forbidden concomitant treatment) and the resulting per protocol (PP) population comprised 56 patients (28 in each group).

Patients’ characteristics were comparable at baseline between the two groups (Table 1). The mean (standard deviation [SD]) age of patients was 65.7 (9.6) years, 44 (65.7%) were women, and 49 (73.1%) presented overweight or class I obesity according to World Health Organisation’s classification^15^. The mean (SD) OA duration was 8.6 (7.1) years and for 40 (67.8%) patients, both knees were affected. Baseline pain scores and Lequesne index were high, indicating that symptomatic OA was associated with pain and very severe disability.

**Table 1:** Baseline characteristics of the Brace and Control Groups (ITT population).

	Brace group (n = 32)	Control group (n = 35)
Age, mean (SD), y	64.8 (11.7)	66.6 (7.2)
Women, No. (%)	24 (75%)	20 (57.1%)
Body mass index, mean (SD), kg/m^2^	29.2 (4.4)	28.1 (5.1)
**Body mass index class** ^**a**^ **, No. (%)**		
Normal	6 (18.8%)	10 (28.6%)
Overweight	10 (31.3%)	13 (37.1%)
Class I obesity	15 (46.9%)	11 (31.4%)
Class II obesity	1 (3.1%)	0 (0.0%)
Class III obesity	0 (0.0%)	1 (2.9%)
Disease duration, mean (SD), y	8.4 (6.8)	8.9 (7.4)
**Kellgren-Lawrence grade, No. (%)**		
Grade II	10 (31.3%)	7 (20.0%)
Grade III	15 (46.9%)	22 (62.9%)
Grade IV	7 (21.9%)	6 (17.1%)
Contralateral knee osteoarthritis, No. (%)	17 (60.7%)	23 (74.2%)
Last 24h-pain (100-mm VAS), mean (SD), mm	63.8 (10.6)	64.7 (13.5)
Pain on motion (100-mm VAS), mean (SD), mm	73.4 (12.7)	71.9 (13.8)
Lequesne index score (0–24), mean (SD)^b^	13.4 (3.7)	12.6 (3.2)

^a^Body mass index class according to World Health Organisation 2006^15^.

^b^Score ranging from 11 to 13 represents very severe handicap; while score ≥14 represents extremely severe handicap.

ITT, Intent-To-Treat; SD, Standard Deviation; VAS, Visual Analog Scale.

### Primary efficacy endpoint

**Figure 3:**
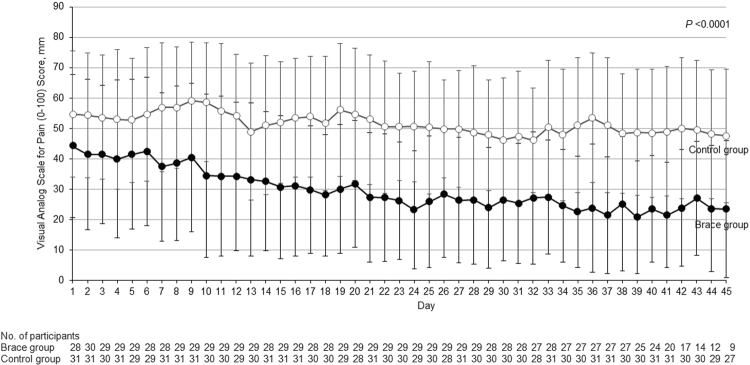
Between-group comparison of change in last 24h-pain scores throughout the study (ITT population). Vertical bars indicate SD for the mean scores. P-value indicates statistical significance between the 2 groups in score change from baseline (Day 0) to Week 6.

Last 24h-pain scores on the ITT population are presented in Table 2. The mean last 24h-pain score significantly decreased over 6 weeks in both groups (Fig. 3). There was a significant between-group difference for change in last 24h-pain score according to estimates of the covariance model at Week 6 (−41.4 mm for the Brace group vs −15.4 mm for the Control group, mean between-group difference −26.0 mm [95% CI −41.6 to −10.3], P < 0.0001) (Table 2). Sensitivity analyzes confirmed the results of the main analysis. Noticeably, pain reduction was observed as soon as Day 10 in the Brace group, while it remained stable in the Control group (Fig. 3).

**Table 2:** Change in study endpoints over 6 weeks between Brace and Control groups (ITT population).

	Brace group (n = 32)			Control group (n = 35)			Between-Group Difference in Change, Mean (95% CI)^b^	P-value
	Mean (SD)		Change, Mean (95% CI)	Mean (SD)		Change, Mean (95% CI)		
	Baseline^a^	Week 6^a^		Baseline^a^	Week 6^a^			
**Primary endpoint**								
Last 24h-pain (100-mm VAS), mm^c^	63.8 (10.6)	22.2 (19.9)	−41.4 (−52.7 to −30.0)	64.7 (13.5)	49.0 (23.4)	−15.4 (−26.2 to −4.6)	−26.0 (−41.6 to −10.3)	<0.0001
Secondary endpoints								
Pain on motion (100-mm VAS), mm^c^	73.4 (12.7)	26.7 (21.5)	−51.9 (−63.6 to −40.2)	71.9 (13.8)	59.7 (22.4)	−19.9 (−31.1 to −8.7)	−32.0 (−48.2 to −15.8)	<0.0001
Lequesne index score (0–24)^d,e^	13.4 (3.7)	7.4 (4.1)	−5.8 (−6.8 to −4.7)	12.6 (3.2)	10.6 (3.7)	−2.3 (−3.3 to −1.3)	−3.5 (−5.0 to −2.0)	<0.0001

^a^Baseline and Week 6 values are measured values.

^b^Between-group differences were calculated using Brace group values minus Control group values.

^c^Change in pain scores are generated from covariance model, with group as fixed factor and baseline value as covariable; missing data were handled according to missing at random assumption.

^d^Change in Lequesne index scores are generated mixed models adjusted with baseline values as covariables, without missing data imputation.

^e^Scores ranging from 5 to 7 represent moderate handicap, from 8 to 10 severe handicap, from 11 to 13 very severe handicap, and score ≥14 extremely severe handicap.

CI, Confidence Interval; ITT, Intent-To-Treat; SD, Standard Deviation; VAS, Visual Analog Scale.

Based on the pre-specified interim analysis, the superiority of the REBEL RELIEVER unloading knee brace combined with usual care compared to usual care alone was demonstrated (P = 0.001). The study was thus prematurely ended in September 2016 after 67 patients had been included.

A significant between-group difference could be observed as soon as Week 2 (−35.5 mm for the Brace group vs −7.6 mm for the Control group, mean between-group difference −26.7 mm [95% CI −35.0 to −18.5], effect size: 1.36, P < 0.001).

### Secondary efficacy endpoints

The results concerning pain on motion and functional disability (Lequesne index) are shown in Table 2. At baseline, Brace and Control groups had similar mean (SD) pain on motion scores and comparable OA high severity and functional disability, as assessed using the Lequesne index. After 6 weeks of treatment, mean pain on motion decrease and Lequesne index score improvement were significantly higher in the Brace group than in the Control group (Table 2). A significant between-group difference was observed from Week 2 for both pain on motion (−45.5 mm for the Brace group vs −12.6 mm for the Control group, mean between-group difference −33.0 mm [95% CI −42.7 to −23.2], P < 0.001) and Lequesne index (−4.7 for the Brace group vs −1.3 for the Control group, mean between-group difference −3.4 [95% CI, −4.9 to −2.0], P < 0.001).

Evolution of disease was assessed by patients on the 7-point scale Patient Global Impression of Change (PGIC; great deal better, better, moderately better, somewhat better, a little better, almost the same, no change), and investigators on the 5-point scale Clinical Global Impression of Improvement (CGI-I; very much improved, much improved, minimally improved, no change, minimally worse). Results from PGIC indicated that 86.2% of patients in the Brace group felt better to a great deal better at Week 6, compared to 43.8% of patients in the Control group who felt almost the same or no change. Similarly, investigators reported much to very much improvement for most patients in the Brace group (82.1%) at Week 6, while they reported no change or worsening in most patients in the Control group (53.2%).

At Week 6, responder rate according to OARSI-Outcome Measures in Rheumatoid Arthritis Clinical Trials (OMERACT) criteria^16^ was significantly higher in the Brace group than in the Control group (72.4% vs 34.4%, P < 0.001).

Analgesic consumption was lower in the Brace group than in the Control group in terms of mean treatment duration throughout the study (1.6 and 8.6 days for NSAIDs and acetaminophen, respectively, in the Brace group, compared to 4.5 and 12.8 days in the Control group). However, these differences were not statistically significant.

### Safety, acceptability and observance

Mean (SD) duration of the REBEL RELIEVER unloading knee brace wearing was 44.0 (10.6) days. Observance to the brace was excellent (>90% of the actual/theoretical wearing days).

Overall, 13 patients (19.4%) reported an adverse event (AE), mainly in the Brace group (10 patients [31.3%]). No serious AE or severe AE were reported. Six patients (18.8%) reported an AE related to the brace, mainly skin irritation or discomfort at brace contact points. No brace-related AE leading to permanent treatment discontinuation was reported.

## Discussion

The purpose of this randomized controlled trial was to assess the additive effect of a REBEL RELIEVER unloading knee brace in conservative treatment of medial OA. Results showed that combination of the REBEL RELIEVER unloading knee brace with usual care was superior to usual care alone in terms of improvement of last 24h-pain, pain on motion, functional disability, disease evolution assessed by both the patient and the investigator, and clinical response rate after a 6-week treatment period. These results are consistent with previous published data suggesting that treatment with unloader braces improves pain and physical function^6,7,10,17–19^.

A 6-week follow-up period was deemed sufficient and clinically relevant to demonstrate the clinical benefit of wearing REBEL RELIEVER unloading knee brace, as it is expected to provide immediate improvement due to its biomechanical effect. Indeed, the brace is designed to apply corrective forces by means of a three-point pressure system. These corrective forces help distribute load away from the damaged compartment, resulting in immediate pain relief. The biomechanical effect of REBEL RELIEVER unloading knee brace has been assessed in OA patients at 2 and 8 weeks after fitting. Significant decrease in knee adduction impulse (KAI) and peak knee adduction moment (KAM) in the second half of the stance phase were observed at both 2 and 8 weeks compared to baseline (36% and 34% reduction in KAI, 26% reduction in second peak KAM at 2 and 8 weeks, respectively; P < 0.05)^20^. The fact that pain decrease was observed as soon as Day 10 in the ROTOR study confirms that REBEL RELIEVER unloading knee brace can be considered as an adjunctive therapy since it can be used for some well-identified daily activities and it does not need to be worn for a long period to show efficacy.

Although systematic review and meta-analysis of studies investigating the efficacy of bracing in knee OA suggested the beneficial effect of this therapy, no formal recommendation on the use of brace could be made mainly because of the poor methodological quality of some studies^13,21–23^. Methodological issues are usually observed in studies evaluating nonpharmacological treatments of knee OA^24^. Indeed, in nonpharmacological treatment assessment, it is often technically or ethically difficult to perform a sham intervention, and the blinding of patients and care providers is frequently impossible, whereas the placebo effect of nonpharmacological treatment is probably important. Moreover, conversely to pharmacological treatment, care providers are an integral part of the effect; the success of the nonpharmacological treatment depends on care providers’ skills, experience, and enthusiasm^24^. As few information exist on the matter of which brace for which OA patient profile, a newly decision tool was recently developed by a French expert panel to help health care providers in OA conservative management (Coudeyre *et al*., in press).

The key strength of this study is the robustness of its design (randomized, comparative study, objective endpoints, data collection methods, missing data [MD] handling, and ITT analysis). Although some specific issues (lack of double-blinding and care provider effect) are difficult to resolve when assessing nonpharmacological treatments, most common methodological issues have been taken into account during study design, study implementation, and data analysis to minimize bias and provide valid and generalizable results. This methodological quality needs to be highlighted as it has been shown that there is limited evidence on the effectiveness of brace treatment for knee OA, mainly because of the lack of well-designed studies^13^. Additional strengths of the ROTOR study are the very good observance to the brace (observance index >90%) and the low number of drops-out. Patient observance has often been reported as an issue in studies investigating the efficacy of bracing^14^. Braces are reportedly difficult to wear for extended periods, due to the degree of force they impart to the limb to alter alignment^21^. Nevertheless, the good short-term compliance observed with the REBEL RELIEVER unloading knee brace suggests long-term compliance sustainability. Level of brace compliance has indeed been directly correlated to the perceived clinical benefit, and patients who described at least a considerable improvement in walking range were more likely (P < 0.001) to continue to use the brace beyond one year^25^.

Despite the good acceptability of the REBEL RELIEVER unloading knee brace reported for included patients, the ROTOR study encountered recruitment issues. The following *a priori* barriers were identified: refusal of patient to wear a brace for aesthetical considerations, poor convincing arguments of the investigators unfamiliar with the unloading brace, common recourse to other therapeutic alternatives such as intra-articular injection of hyaluronic acid, and wish for short-term symptom relief. The suspected excellent outcomes led to trigger an interim analysis when half of patients initially planned to be included ended follow-up. All statistical precautions were taken to provide robust results: the conservative Haybittle-Peto method was used to handle the multiplicity issue due to the interim analysis. This allowed maintaining the overall experimental wide alpha level at 0.05. Of note, post-hoc analysis based on the observed effect size of 1.36 and actual sample size gave a power value of 0.96.

Assessment of physical function in patients with OA is challenging as many factors are associated with disability in symptomatic knee OA. In daily practice, pain, functional impairment and quality of life are the most frequent parameters used in the management of OA. In clinical trials, according to European Medicines Agency’s guidelines, pain attributable to the target joint is recommended as the primary endpoint, while functional disability should be included as co-primary endpoint^26^. The OARSI and OMERACT also support the use of outcome measures that evaluate both pain and function^16^. The results of this trial show a reduction of more than 40 mm in last 24h-pain score on a 100-mm VAS, and 50 mm in pain on motion score in the Brace Group compared to baseline. The OMERACT-OARSI responder rate is of 75%, which is superior to that observed in previous clinical trials with others products. According to the recent American Medical Society for Sport Medicine (AMSSM) review, patients receiving hyaluronic acid were 15% more likely to respond to treatment on OMERACT-OARSI criteria while the intra-articular corticosteroid injections were not associated with an improved OARSI responder rate. This suggests that biomechanical intervention might be a powerful strategy in OA management and unloading the affected compartment may lead to a rapid and more effective reduction of inflammatory local process^27^.

Patient-reported outcome measures are being developed for more relevant assessments of pain management. Among them, the concepts of minimal clinically important improvement (MCII, “feeling better”) and patient acceptable symptomatic state (PASS, “feeling well”) are supported by the OMERACT^28,29^. Thus, the rate of patients with an improved condition and the rate of patients in an acceptable condition at the end of the trial are reported. Both criteria are useful from a clinical point of view. Such variables may be included in further studies investigating the effect of brace in the management of knee OA.

In conclusion, the 6-week treatment with the REBEL RELIEVER unloading knee brace combined with usual care was demonstrated safe and superior to usual care alone in relieving global pain and improving motion. Hence, combining a REBEL RELIEVER unloading knee brace with usual care is a powerful therapeutic strategy to reduce pain and improve function in medial knee osteoarthritis patients. This warrants further investigations in larger or longer clinical trials, in terms of OA progression.

## Methods

### Study Design

The ROTOR study was a multicenter, prospective, randomized, controlled, open-label, two-arm, parallel-group, Phase III trial carried out in France by nine private-practice physicians managing OA either in primary or secondary care (including general practitioners, rheumatologists, orthopedic surgeons, and specialists in physical medicine and rehabilitation) and one hospital-based physician from November 2013 through July 2016.

The study was carried out in accordance with principles of the Declaration of Helsinki and Good Clinical Practices. The study was approved by the Ethics Committee (*Comité de Protection des Personnes*, CPP) of Ile de France V and the French National Public Health Agency (ANSM). Participants provided written informed consent prior to their participation in the study. The study is registered at ClinicalTrials.gov (NCT02021136 - Registration date 27^th^ December 2013).

### Patients

The study enrolled adult patients with body mass index ≤35 kg/m^2^, presenting with symptomatic medial knee OA defined as pain while walking for 30 days over the past 2 months prior to inclusion, and global knee pain over the last 24 h ≥40 mm (using a 100-mm visual analogic scale [VAS]) on the day of inclusion. Diagnosis of medial knee compartment OA was performed according to American College of Rheumatology criteria^30^, and based on radiological findings within the previous 24 months (Kellgren-Lawrence grade II-IV for the medial compartment ± Kellgren-Lawrence grade I for the lateral compartment).

Patients with symptomatic OA of the patellofemoral knee compartment (radiologically-diagnosed), septic arthritis, metabolic arthropathies, inflammatory rheumatic diseases, synovitis needing aspiration, contralateral knee OA needing intra-articular corticosteroids, varicous veins or venous reflux disease, lower limbs sensory disorders, lower limbs arteritis, history of intra-articular injection of hyaluronic acid in the evaluated knee or intra-articular corticosteroids administration in either knee within the last month, or history of taking opioids, corticosteroids, non-steroidal anti-inflammatory drugs (NSAIDs) or analgesics within the last 48 h were excluded.

The knee that met the previously described inclusion and exclusion criteria was selected as the study knee for outcome measures. When both knees met the criteria, the study knee was defined as the most affected one.

### Procedures

After the informed consent was given, baseline measurements were made and patients were randomly assigned to treatment group in a 1:1 ratio using an interactive voice/web response system (IVRS/IWRS) together with a minimization algorithm to ensure that the groups were balanced within sites. The follow-up assessments took place after 2 and 6 weeks of brace wearing (Week 2 and Week 6).

### Interventions

All patients received usual care, consisting of analgesics (acetaminophen and NSAIDs), daily exercise program as recommended by the French Society of Rheumatology^31^, and patient information, as per OARSI’s guidelines^5^. In addition, patients in the Brace group had to wear the REBEL RELIEVER unloading knee brace (Thuasne, Fig. 1) for at least 6 h daily for 6 weeks. Initial fitting of the brace was performed by an orthopedist-orthotist, who made any necessary adjustments to create a base level of corrective force and gave instructions to the patients.

### Outcomes

The primary outcome was global last-24h knee pain relief (measured using a 100-mm VAS) after a 6-week treatment period. Secondary outcomes included evolution of pain on motion (100-mm VAS), functional disability (Lequesne index)^32^, PGIC, CGI-I, responder rate according to OARSI- OMERACT criteria (which defined clinical response as pain decrease [VAS] ≥50% and ≥20 mm or Lequesne index decrease ≥50%)^16^, analgesic consumption, as well as the REBEL RELIEVER safety and acceptability. Finally, observance was assessed using patient’s diary, which collected daily data on brace wearing (yes/no); duration of brace wearing (full day/half day/1 to 2 hours during activities), reason of non-wearing (no pain/too constraining/contact allergy/walk disturbance/unsteadiness), daily exercise program (yes/no), analgesics intake (yes/no), and type of analgesics administered (acetaminophen/aspirin/NSAIDs/other).

### Sample size

The primary objective was to evaluate the superiority of the REBEL RELIEVER unloading knee brace in combination with usual care on usual care alone in terms of global last-24h knee pain relief after a 6-week treatment period. A sample size of 50 patients per treatment group was estimated to be sufficient to detect a difference of 10-mm in last 24h-pain score with 80% power (α = 0.05) and a SD of 20 mm. Assuming 15% of MD or premature discontinuation, the number of patients was increased to 59 patients per treatment group to keep power of 80% for analysis on PP population. An inter-group difference of 10-mm for pain score was considered as clinically relevant.

### Statistical analyses

The ITT population included all randomized patients. The safety population included all randomized patients of Control group and all randomized patients of Brace group bearing at least once the brace. The PP population included all patients of the ITT population without any major protocol deviation, exposed for at least 30 days to the brace, and assessed for primary endpoint at 6 weeks.

Analyses were carried out using SAS version 9.4 (SAS Institute Inc) using an ITT approach. Two-sided P-value of 0.05 was considered statistically significant unless otherwise specified. The primary endpoint was analyzed using a covariance model, with group as fixed factor and baseline value as covariable. The main analysis of primary endpoint was conducted on the ITT population by handling MD according to missing at random assumption. Sensitivity analyses were conducted on the PP population and on the ITT population without handling MD. Secondary outcome assessments at Week 2 and Week 6 (pain on motion, Lequesne index) were analyzed using mixed model of repeated measures (MMRM), with baseline values as covariables. Between-group comparisons of PGIC, CGI-I and responder rate were performed using Wilcoxon or Fisher test, as appropriate.

Based on the hypothesis of a much larger efficacy than initially anticipated, an interim analysis was submitted and approved by the Ethics Committee (CPP of Ile de France V) on October 2015. It has been conducted when about 50% of randomized patients had completed the study. The interim analysis was performed using the Haybittle-Peto monitoring boundaries for extreme positive results. These boundaries are conservative and require small P-values for early stopping of the trial without undermining its validity and integrity^33,34^. Thus, P-value was 0.001 for the primary outcome with the aim to prematurely stop the trial in case of strong additive clinical benefit of the REBEL RELIEVER unloading knee brace.

### Data availability

The full dataset is available from the corresponding author at philippe.thoumie@aphp.fr on reasonable request.

## References

[1] Felson DT (1995). The incidence and natural history of knee osteoarthritis in the elderly. The Framingham Osteoarthritis Study. Arthritis Rheum..

[2] Peat G, McCarney R, Croft P (2001). Knee pain and osteoarthritis in older adults: a review of community burden and current use of primary health care. Ann. Rheum. Dis..

[3] Guillemin F (2011). for the 3000 Osteoarthritis group. Prevalence of symptomatic hip and knee osteoarthritis: a two-phase population-based survey. Osteoarthritis Cartilage..

[4] McAlindon TE, Snow S, Cooper C, Dieppe PA (1992). Radiographic patterns of osteoarthritis of the knee joint in the community: the importance of the patellofemoral joint. Ann. Rheum. Dis..

[5] McAlindon TE (2014). OARSI guidelines for the non-surgical management of knee osteoarthitis. Osteoarthritis Cartilage..

[6] Matsuno H, Kadowaki KM, Tsuji H (1997). Generation II knee bracing for severe medial compartment osteoarthritis of the knee. Arch. Phys. Med. Rehabil..

[7] Kirkley A (1999). The effect of bracing on varus gonarthrosis. Bone Joint Surg. Am..

[8] Birmingham TM, Kramer JF, Kirkley A, Inglis JT, Spaulding SJ, Vandervoort AA (2001). Knee bracing for medial compartment osteoarthritis: effects on proprioception and postural control. Rheumatology..

[9] Barnes CL, Cawley PW, Hederman B (2002). Effect of CounterForce brace on symptomatic relief in a group of patients with symptomatic unicompartmental osteoarthritis: a prospective 2-year investigation. Am. J. Orthop. (Belle Mead NJ)..

[10] Brouwer RW, van Raaij TM, Verhaar JA, Coene LN, Bierma-Zeinstra SM (2006). Brace treatment for osteoarthritis of the knee: a prospective randomized multi-centre trial. Osteoarthritis Cartilage..

[11] Van Raaij TM, Reijman M, Brouwer RW, Bierma-Zeinstra SM, Verhaar JA (2010). Medial knee osteoarthritis treated by insoles or braces: a randomized trial. Clin. Orthop. Relat. Res..

[12] Wilson B, Rankin H, Barnes CL (2011). Long-term results of an unloader brace in patients with unicompartmental knee osteoarthritis. Orthopedics..

[13] Duivenvoorden T, Brouwer RW, van Raaij TM, Verhagen AP, Verhaar JA, Bierma-Zeinstra SM (2015). Braces and orthoses for treating osteoarthritis of the knee (Review). Cochrane Database Syst. Rev..

[14] Beaudreuil J (2016). Orthoses for osteoarthritis: a narrative review. Ann. Phys. Rehabil. Med..

[15] World Health Organisation “BMI Classifications”, http://www.who.int/bmi/index.jsp?introPage=intro_3.html (2006).

[16] Pham T (2004). OMERACT-OARSI initiative: Osteoarthritis Research Society International set of responder criteria of osteoarthritis clinical trials revisited. Osteoarthritis Cartilage..

[17] Lindenfeld TN, Hewett TE, Andriacchi TP (1997). Joint loading with valgus bracing in patients with varus gonarthrosis. Clin. Orthop. Rel. Res..

[18] Hillstrom HJ (2000). Assessment of conservative realignment therapies for the treatment of varus knee osteoarthritis: biomechanics and joint pathology. Gait Posture..

[19] Draganich L, Reider B, Rimington T, Piotrowski G, Mallik K, Nasson S (2006). The effectiveness of self-adjustable custom and off-the-shelf bracing in the treatment of varus gonarthrosis. J. Bone Joint Surg. Am..

[20] Lamberg EM, Steb R, Werner M, Kremenic I, Penna J (2016). The 2- and 8-week effect of decompressive brace use in people with medial compartment knee osteoarthritis. Prosthet. Orthot. Int..

[21] Ramsey DK, Russel ME (2009). Unloader braces for medial compartment knee osteoarthritis: implications on mediating progression. Sports Health..

[22] Moyer RF, Birmingham TB, Bryant DM, Giffin JR, Marriott KA, Leitch KM (2015). Valvus bracing for knee osteoarthritis: a meta-analysis of randomized trials. Arthritis Care Res. (Hoboken)..

[23] Petersen W (2016). Biomechanical effect of unloader braces for medial osteoarthritis of the knee: a systematic review (CRD 42015026136). Arch. Orthop. Trauma Surg..

[24] Boutron I, Tubach F, Giraudeau B, Ravaud P (2003). Methodological differences in clinical trials evaluating nonpharmacological and pharmacological treatments of hip and knee osteoarthritis. JAMA..

[25] Squyer E, Stamper DL, Hamilton DT, Sabin JA, Leopold SS (2013). Unloader knee braces for osteoarthritis: do patients actually wear them?. Clin. Orthop. Relat. Res..

[26] European Medicines Agency. Guideline on clinical investigation of medicinal products used in the treatment of osteoarthritis, http://www.ema.europa.eu/docs/en_GB/document_library/Scientific_guideline/2009/09/WC500003440.pdf (CPMP/EWP/784/97 Rev. 1). January 2010.

[27] Trojian TH, Concoff AL, Joy SM, Hatzenbuehler JR, Saulsberry WJ, Coleman CI (2016). AMSSM scientific statement concerning viscosupplementation injections for knee osteoarthritis: importance for individual patient outcomes. Br. J. Sports Med..

[28] Tubach F (2007). Minimally clinically important improvement and patient acceptable symptom state for subjective outcome measures in rheumatic disorders. J. Rheumatol..

[29] Tubach F (2012). Minimum clinically important improvement and patient acceptable symptom state in pain and function in rheumatoid arthritis, ankylosing spondylitis, chronic back pain, hand osteoarthritis, and hip and knee osteoarthritis: Results from a prospective multinational study. Arthritis Care Res. (Hoboken)..

[30] Altman R (1986). Diagnostic and Therapeutic Criteria Committee of the American Rheumatism Association. Development of criteria for the classification and reporting of osteoarthritis: classification of osteoarthritis of the knee. Arthritis Rheum..

[31] French Society of Rheumatology. Exercises for knee osteoarthritis, http://public.larhumatologie.fr/fiches-dauto-exercice-arthrose.

[32] Lequesne M, Mery C, Samson M, Gérard P (1987). Indexes of severity for osteoarthritis of the hip and knee. Validation–value in comparison with other assessment tests. Scand. J. Rheumatol..

[33] Haybittle JL (1971). Repeated assessment of results in clinical trials of cancer treatment. Brit. J. Radiol..

[34] Peto R (1976). Design and analysis of randomized clinical trials requiring prolonged observation of each patient: I. Introduction and design. Br. J. Cancer..

